# Complete genomes of three endophytic *Pseudomonas putida* isolates from *Agave salmiana*, assembled using Illumina and PacBio sequencing

**DOI:** 10.1128/mra.01351-25

**Published:** 2026-01-27

**Authors:** Martha Giles-Gómez, Diego Guerrero Corona, Elizabeth Martínez García, Francisco Bolívar, Adelfo Escalante

**Affiliations:** 1 Departamento de Biología, Facultad de Química, Universidad Nacional Autónoma de México, Ciudad Universitaria 7180, Ciudad de México, México; 2 Departamento de Ingeniería Celular y Biocatálisis, Instituto de Biotecnología. Universidad Nacional Autónoma de México 7180, Cuernavaca, Morelos, México; University of Manitoba, Winnipeg, Manitoba, Canada

**Keywords:** *Pseudomonas putida*, endophytes, *Agave salmiana*, complete genome, Illumina NovaSeq, PacBio

## Abstract

We report the complete genome sequences of three endophytic *Pseudomonas putida* isolates from *Agave salmiana*, assembled using Illumina NovaSeq and PacBio HiFi sequencing. Genome sizes were 6,282,325 bp (PP_P5), 6,511,513 bp (PP_P9), and 6,284,694 bp (PP_P43), respectively.

## ANNOUNCEMENT

Members of the genus *Pseudomonas* are widespread plant-associated bacteria, including strains that act as endophytes with potential roles in plant growth promotion, stress tolerance, and biocontrol. Endophytic *Pseudomonas* spp. have previously been reported in agave species, highlighting their ecological relevance and possible contribution to plant physiology (1–3). Here, we report the complete genome sequences of three endophytic *Pseudomonas putida* strains isolated from *Agave salmiana* (4), sequenced using the Illumina NovaSeq and PacBio Sequel platforms. This announcement represents the first report of complete genome sequences of endophytic *Pseudomonas* strains from agave. Their characterization provides a foundational resource for future studies on agave-endophyte interactions and the potential development of microbial inoculants.

Endophytic *Pseudomonas* strains were isolated from the embryonic floral peduncle that surrounds the central leaves (floral peduncle phyllotaxy zone) of a mature (5–6 years old) *A. salmiana* (maguey) plant sampled in Huitzilac, Morelos, Mexico (19°01′42″N 99°16′02″W), following the methodology described previously (4). Colonies grown on DIFCO Tryptic Soy Agar supplemented with 0.5 μg/mL cycloheximide (Sigma) were purified by the streak-plate method, and Gram staining was performed. Isolated strains were maintained in 40% glycerol stocks at −70°C. Bacterial DNA was extracted from an overnight culture in DIFCO Tryptic Soy Broth at 30°C without shaking, using the DNeasy UltraClean Microbial Kit (QIAGEN, Hilden, Germany), according to the manufacturer’s instructions. DNA quality was verified by 1% agarose gel electrophoresis.

Ten micrograms of genomic DNA of each isolate was sent to Novogene Co., Ltd (Sacramento, CA) for library construction, quality control, and sequencing by Illumina NovaSeq X Plus series and PacBio Sequel II/IIe systems. A genomic DNA library for Illumina sequencing was prepared using the Rapid Plus DNA Library Prep Kit (ABclonal) according to the manufacturer’s standard protocol. The PacBio HiFi library was prepared using the SMRTbell Prep Kit 3.0 (Pacific Biosciences) according to the manufacturer’s standard protocol.

Illumina NovaSeq sequencing produced 9,946,938, 14,183,022, and 12,977,976 paired-end reads for strains PP_P5, PP_P9, and PP_P43, respectively. PacBio Sequel sequencing yielded 3,757,916,046, 3,728,124,989, and 4,261,168,756 bp of HiFi reads for strains PP_P5, PP_P9, and PP_P43, respectively. Read quality was assessed using FastQC v0.12.1 for Illumina data (https://www.bioinformatics.babraham.ac.uk/projects/fastqc). PacBio read quality was assessed using NanoPlot v1.46.1 (5). Illumina reads were trimmed and filtered using standard quality-control tools, and assemblies were polished with Pilon v1.24 (6). PacBio HiFi reads were assembled with Hifiasm v0.25.0 (7). The assembly obtained with PacBio was refined with trimmed and filtered Illumina reads using the Pilon v1.24 parameters (6), yielding a single contig of 6,282,417 bp for PP_P5, a single contig of 6,317,734 bp for PP_P9, and a single contig of 6,284,694 bp for PP_P43.

Gene annotation was performed with the NCBI Prokaryotic Genome Annotation Pipeline (8). Species identification was confirmed as *Pseudomonas putida* by BLAST v2.11.0 (9). Assembly quality was evaluated using QUAST v5.2.0 (10) with the *P. putida* strain NBRC 14164 reference genome GCF_000412675.1 from NCBI. Table 1 summarizes the genome assemblies. All software tools were run using default parameters, except where otherwise noted.

**TABLE 1:** Accession numbers and sequence features of assembled genomes of endophytic *Pseudomonas putida* strains isolated from *Agave salmiana*

Strain	PacBio raw reads	PacBio N50 (bp)	Illumina raw reads	SRA number	Total assembly length (bp)	Coverage	Total CDS	GC content (%)	Contig/replicon	GenBank accession number	Contig length (bp)
PP_P5	220,325	17,497	9,946,938	SRX30911656	6,282,417	598.16×	5,620	62.12	Contig_1/closed chromosome	JBRUQK000000000	6,282,417
PP_P9	217,087	17,716	14,183,022	SRX30911658	6,317,734	572.54×	5,867	61.97	Contig_1/closed chromosome	JBRUQJ000000000	6,317,734
PP_P43	248,702	17,381	12,977,976	SRX30911660	6,284,694	678.02×	5,616	62.12	Contig_1/closed chromosome	JBRUQI000000000	6,284,694

## Data Availability

This Whole Genome Shotgun project has been deposited at DDBJ/ENA/GenBank under BioProject PRJNA1021245. *Pseudomonas putida* isolates were deposited under Biosample SAMN52095935 (*P. putida* P5), Biosample SAMN52095936 (*P. putida* P9), and Biosample SAMN52095937 (*P. putida* P43). The versions described in this announcement are JBRUQK000000000, JBRUQJ000000000, and JBRUQI000000000. Accession and Illumina SRA numbers for each strain are shown in Table 1.
